# Aspirin Dose and Treatment Outcomes in Kawasaki Disease: A Historical Control Study in Japan

**DOI:** 10.3389/fped.2020.00249

**Published:** 2020-05-14

**Authors:** Yu Ito, Takuya Matsui, Kota Abe, Takafumi Honda, Kumi Yasukawa, Jun-ichi Takanashi, Hiromichi Hamada

**Affiliations:** ^1^Department of Pediatrics, Tokyo Women's Medical University Yachiyo Medical Center, Chiba, Japan; ^2^Department of Pediatric Intensive Care, Tokyo Women's Medical University Yachiyo Medical Center, Chiba, Japan

**Keywords:** aspirin, treatment, coronary artery, Kawasaki disease (KD), immunoglobulin

## Abstract

Aspirin has been used as a concomitant drug in the treatment of Kawasaki disease (KD). In recent years, there has been discussion concerning whether high-dose aspirin is appropriate for treatment in the acute phase of KD. We retrospectively investigated the incidence of coronary artery abnormalities (CAAs) and the antipyretic effect of 30 to 50 mg/kg/day aspirin, the minimum and the maximum approved doses in Japan. This was a single-center, non-randomized, retrospective, historical cohort study. Patients were routinely treated with 50 mg/kg/day aspirin (50-mg Group) between 2007 and April 2014, and with 30 mg/kg/day aspirin (30-mg Group) between May 2014 and 2016. All patients were given initial and, if necessary, subsequent intravenous immunoglobulin (IVIG) 2.0 g/kg. The primary endpoint was incidence of CAAs defined as a CA diameter with a Z score ≥2.5 at treatment week 4. The secondary endpoint was incidence of further treatment. Incidences were compared using inverse probability weighting analysis adjusting for age, sex, and risk scores. In 587 patients, there was no significant difference in incidence of CAAs (odds ratio in 30-mg Group 0.769, 95% confidence interval (CI): 0.537–1.101, *p* = 0.151). Risk of further treatment after the first IVIG in the 30-mg Group was significantly higher than that in the 50-mg Group (odds ratio 1.379, 95% CI: 1.051–1.811, *p* = 0.021). Although this study has some limitations, the findings suggest that aspirin 50 mg/kg/day may have no significant effect on improving incidence of CAAs compared with 30 mg/kg/day but may have a lower rate of further treatment.

## Introduction

Kawasaki disease (KD), which is an acute vasculitis in young children and causes coronary artery abnormalities (CAAs) such as coronary artery aneurysm and dilation, is the leading cause of acquired heart disease in children in developed countries (1, 2). In the five decades since the initial recognition of KD, the standard treatment is a combination of intravenous immunoglobulin (IVIG) and acetylsalicylic acid (ASA) (3). High-dose (80–100 mg/kg) and medium-dose (30–50 mg/kg) ASA have been recommended as standard treatment during the acute febrile phase by the American Heart Association (AHA) and Japanese Society of Pediatric Cardiology and Cardiac Surgery (JSPCCS) (4, 5).

ASA was first confirmed to be effective for KD in the latter half of the 1970s in Japan. That prospective study compared ASA, flurbiprofen, and prednisolone plus dipyridamole (6). At 1 month after onset, ASA could not prevent the incidence of CAAs, but at 1 year after onset, only 1% of ASA-treated patients had CAAs. These results were far better compared with 12% in the flurbiprofen-treated group and 9% in the prednisolone plus dipyridamole-treated group, indicating the superiority of ASA (4). Mortality dropped from 1.7 to 0.2–0.3% after the ASA treatment was adopted (7). A US study prior to the spread of immunoglobulin therapy has also shown that ASA suppresses the development of CAAs (8).

In the 1990s, IVIG became the mainstay of treatment, and when the development of CAAs was the final prognosis, the impact of ASA became less pronounced (9, 10). In recent years, some retrospective studies have raised the question of whether high-dose and medium-dose ASA would be appropriate for KD treatment in terms of risk-benefit (11–14). Starting treatment with high or medium doses of ASA (≥30 mg/kg/day) was found not to prevent CAAs (11–14).

In Japan, the Pharmaceuticals and Medical Devices Agency has approved ASA 30–50 mg/kg/day for KD. However, no studies have investigated ASA dose and outcomes in KD within the recent IVIG era in Japan. For 11 years now we have treated KD patients with a unified treatment protocol of IVIG and ASA as first-line therapy. Between January 2007 and April 2014, patients received 50 mg/kg/day ASA. After May 2014, patients were given 30 mg/kg/day ASA. In this study, we retrospectively investigated differences in the incidence of CAAs as well as the antipyretic effect between historical control 50 mg/kg/day and 30 mg/kg/day ASA at a single center in Japan.

## Methods

This single-center, non-randomized, retrospective, historical cohort study had a target population that comprised 587 children between 0 and 12 years of age diagnosed with complete or incomplete KD between 2007 and 2016 at Tokyo Women's Medical University Yachiyo Medical Center, Chiba, Japan. Between January 2007 and April 2014, patients received 50 mg/kg/day ASA. Between May 2014 and September 2016, patients were given 30 mg/kg/day ASA.

Inclusion criteria were children with a diagnosis of KD who were treated with IVIG and ASA. Given that the diagnosis of KD cannot be confirmed definitively, patients were eligible if the clinical suspicion of KD was such that treatment with IVIG was deemed clinically indicated.

Exclusion criteria were as follows: patients for whom a final diagnosis of KD was clearly ruled out; patients who had preexisting CAAs before treatment diagnosed at bedside according to the Japanese Ministry of Health, Labour and Welfare criteria (15), which are based on absolute values (≥3 mm in children <5 years old, ≥4 mm in children ≥5 years old); patients who had received any treatment for KD at other medical hospital before arriving at our institution; patients who were enrolled in a clinical study and had received cyclosporine with IVIG plus aspirin as first-line therapy (16).

Research Ethics Board approval was granted at Tokyo Women's Medical University (number 3636-R2). Only anonymous retrospective data were obtained for this study, and the requirement for patient consent was waived by the Research Ethics Board.

Patients received initial IVIG 2.0 g/kg for 24 h and ASA. An additional dose of IVIG 2.0 g/kg was administered to patients who were non-responders, defined as body temperature ≥37.5°C at 48 h after initiation of primary therapy, and those who had KD relapse, defined as a return of fever (≥37.5°C) without another likely source after a ≥48-h afebrile period from the initiation of primary therapy. Patients whose body temperature did not drop to below 37.5°C at 24 h after the initiation of the second dose of IVIG received cyclosporine or/and a third dose of IVIG as third-line therapy.

Between January 2007 and April 2014, patients received 50 mg/kg/day ASA (ASA 50-mg Group). Between May 2014 and September 2016, patients were given 30 mg/kg/day ASA (ASA 30-mg Group). At 48 h after defervescence, aspirin dose was reduced to 5 mg/kg/day in all patients. Low-dose ASA was continued for 4 to 6 weeks.

The primary endpoint was the incidence of CAAs at treatment week 4. CAAs were diagnosed according to Z scores in Japanese children—echocardiographic measurements of the internal diameter normalized by body surface area—of coronary artery dimensions for the following three segments: proximal right coronary artery, left main coronary artery, and proximal left anterior descending artery (17). The maximum Z score was defined by the largest Z score in the three segments. CAAs were defined as present when the maximum Z score was ≥2.5, according to 2017 AHA guidelines. The value of the Z score was retrospectively calculated using absolute diameters (mm) in the echo reports at 1 month after onset.

The secondary endpoint was the incidence of second- and third-line therapy in the two treatment groups.

Adverse events were extracted from elevated liver enzymes, anemia, and bleeding. The incidences of elevated liver enzymes in ASA 50-mg Group and ASA 30-mg Group were compared. Elevated liver enzymes was defined as aspartate aminotransferase >50 U/L and alanine aminotransferase >50 U/L in the convalescent phase of KD. Differences in hemoglobin levels before and after the first IVIG dose were compared between the two groups. The incidence of severe bleeding or digestive symptoms suggesting gastritis or gastric ulcer was also compared between the two groups.

### Statistical Analysis

We used a non-inferiority design to assess whether the risk ratio of CAAs between treatment groups would remain within the 95% confidence interval (CI) of a predefined clinically acceptable margin. In primary endpoint and secondary endpoint, data were adjusted by inverse probability weighting method for age, sex, and risk scores, and logistic regression was performed. A regression coefficient test was performed using the Wald test, and a *p* < 0.05 was considered significant.

Items under patient characteristics were assessed using Welch's *t*-test, Pearson's χ^2^ test, or a two-sided *t*-test. The incidence of adverse events was assessed using Fisher's exact test. Two-tailed *P* < 0.05 was considered statistically significant.

## Results

A total of 655 patients were identified and screened for eligibility. Of them, 68 were excluded for the following reasons: 8 patients had CAAs before treatment; 39 patients had received any treatment for KD at other hospitals before referral to our institution; and 21 patients were enrolled in a clinical study and received cyclosporine with IVIG plus ASA as first-line therapy. The 587 remaining patients were included in the final analysis. Among them, 414 patients were routinely prescribed 50 mg/kg/day ASA between January 2007 and April 2014 (ASA 50-mg Group). The remaining 173 patients were routinely prescribed 30 mg/kg/day ASA between May 2014 and September 2016 (ASA 30-mg Group).

(Table 1) presents the characteristics of the study population. Both groups were similar in age, sex, risk scores (Kobayashi score for predicting resistance to IVIG) (18), number of days of fever before the first IVIG dose, C-reactive protein before treatment, and hemoglobin levels.

**Table 1:** Patient characteristics.

**Characteristic**	**ASA 50-mg Group *n* = 414**	**ASA 30-mg Group *n* = 173**	***p*-value**
Age, years	2.6 (1.9)	2.7(2.1)	0.628*^a^
Age <1 year at diagnosis, %	22.2%	24.2%	0.680*^b^
Male sex, %	57.6%	54.0%	0.376*^b^
Risk score (Kobayashi)	3.5 (2.3)	3.6 (2.6)	0.837*^a^
Day of illness at first IVIG treatment, days	5.2 (1.6)	5.1 (1.3)	0.339*^a^
Hemoglobin, g/dL	11.3 (1.2)	11.6 (1.0)	0.830*^a^
White blood cell count, ×103/μL	13.9 (4.4)	13.9 (4.6)	0.851*^a^
Neutrophils ≥80% at diagnosis	23.7%	23.7%	0.423*^c^
Platelet count, ×104/μL	33.2 (10.2)	32.1 (9.3)	0.053*^c^
C-reactive protein, mg/dL	7.5 (4.6)	8.3 (4.7)	0.373*^c^
Albumin, g/dL	3.9 (0.3)	3.9 (0.4)	0.842*^c^
Na, mEq/L	134 (2.6)	134 (2.9)	0.410*^c^
Aspartate aminotransferase, U/L	104 (215)	121 (240)	0.183*^c^

*Data are shown as the mean (SD) or percentage*.

*a *Welch's t-test*.

*b *Pearson's χ^2^ test*.

*c *t-test, two-sided*.

Our primary outcome, the presence of CAAs, was observed in 70 patients (12%) in total. Of these, number of patients with small CAAs (z score ≥2.5 but <5), medium-sized CAAs, and large-sized CAAs in each treatment group are shown in (Table 2A). (Table 2B) presents the proportion of patients with CAAs according to treatment groups as well as the unadjusted and adjusted risk ratios. The incidence of CAAs was not significantly different between the ASA 50-mg Group and the ASA 30-mg Group.

**Table 2:** Coronary artery outcomes.

**(A)** Numbers and percentages of patients with CAAs.				
	**ASA 50-mg Group** ***n*** **=** **414 number (%)**		**ASA 30-mg Group** ***n*** **=** **173 number (%)**	
All	52 (12.6)		18 (10.4)	
Small (Z ≥2.5, Z <5.0)	50 (12.1)		14 (8.1)	
Medium (Z ≥5.0, Z <10.0)	1 (0.2)		3 (1.7)	
Large (Z ≥10.0)	1 (0.2)		1 (0.6)	
**(B)** Unadjusted and adjusted odds ratios.				
	**Unadjusted**		Adjusted*	
	***p*****-value**	**Odds ratio (95% CI)**	***p*****-value**	**Odds ratio (95% CI)**
ASA 30-mg Group	0.346	0.760 (0.430–1.345)	0.151	0.769 (0.537–1.101)
Age <12 months	0.006	2.148 (1.243–3.711)	0.003	1.844 (1.237–2.747)
Sex=M	0.935	0.979 (0.592–1.619)	0.455	0.872 (0.609–1.249)
Risk score (Kobayashi)	0.966	0.979 (0.376–2.553)	0.074	1.897 (0.939–3.832)

* *Inverse probability weighting analysis*.

For the secondary endpoint, requiring further treatment after the first IVIG dose was observed in 143 patients (24%) in total. The risk was significantly higher in the ASA 30-mg Group than in the ASA 50-mg Group (Tables 3A,B).

**Table 3:** Patients needing second IVIG and third-line treatment.

**(A)** Numbers of patients needing second IVIG and third-line treatment					
	**ASA 50-mg Group** ***n*** **=** **414 number (%)**			**ASA 30-mg Group** ***n*** **=** **173 number (%)**	
Second IVIG	95 (22.9)			48 (27.7)	
Third-line treatment	21 (5.0)			13 (7.5)	
**(B)** Incidence of patients needing second IVIG and third-line treatment.					
		**Unadjusted**		Adjusted*	
		***p*****-value**	**Odds ratio (95% CI)**	***p*****-value**	**Odds ratio (95% CI)**
Second IVIG	ASA 30-mg Group	0.112	1.405 (0.923–2.139)	0.021	1.379 (1.051–1.811)
	Age <12 months	0.006	0.457 (0.263–0.795)	0.003	0.579 (0.402–0.835)
	Sex = M	0.066	1.456 (0.975-2.175)	0.079	1.281 (0.971-1.689)
	Risk score (Kobayashi)	<0.001	9.427 (4.105–21.647)	<0.001	9.453 (5.345–16.718)
Third-line treatment	ASA 30-mg Group	0.264	1.534 (0.725–3.246)	0.106	1.512 (0.916–2.495)
	Age <12 months	0.288	0.554 (0.186–1.647)	0.597	0.832 (0.422–1.642)
	Sex=M	0.140	1.780 (0.827-3.830)	0.041	1.716 (1.023-2.878)
	Risk score (Kobayashi)	<0.001	135.399 (17.170–1067.709)	<0.001	256.728 (58.648–1123.812)

* *Inverse probability weighting analysis*.

For safety outcomes, the incidences of elevated liver enzymes at convalescent phase were similar between the two groups (Table 4A). There was no significant difference between the two groups in the reduction of hemoglobin before and after the first IVIG (Table 4B). No severe hemorrhagic complications nor digestive symptoms were observed in either group.

**Table 4A:** Safety outcomes.

**(A)**. Hepatic dysfunction.				
		**ASA 50-mg/kg/day Group** ***n*** **=414, mean** **±** **SD**	**ASA 30-mg/kg/day Group n=173, mean** **±** **SD**	***p*****-value**
Incidence of patients with hepatic dysfunction*^a^, %		15%	17%	0.078*^b^
Liver enzymes at convalescent phase	Aspartate aminotransferase	61.6 ± 82.1	54.2 ± 60.5	0.228*^c^
	Alanine aminotransferase	43.7 ± 66.8	33.7 ± 52.7	0.057*^c^

*a *Defined as aspartate aminotransferase >50 U/L and alanine aminotransferase >50 U/L at convalescent phase*.

*b *Pearson's χ^2^ test*.

*c *Welch's t-test*.

**Table 4B:** 

**(B)**. Hemoglobin.			
	**ASA 50-mg Group, mean (SD)/percentage**	**ASA 30-mg Group, mean (SD)/percentage**	***p*****-value**
Pre IVIG, g/dL	11.3 ± 1.2	11.6 ± 1.0	0.045*^a^
Post IVIG, g/dL	10.9 ± 1.2	11.1 ± 1.4	0.833*^a^
Difference (post-pre), g/dL	−0.4 ± 1.1	−0.5 ± 1.5	*p* = 0.895*^a^

*a *Welch's t-test*.

## Discussion

The results of our study suggest that using 30 mg/kg/day ASA, which is the lower limit of the approved dose for acute KD in Japan, is not inferior to 50 mg/kg/day ASA, which is the upper limit dose, in preventing CAAs. However, we observed a significant effect of ASA dose on treatment resistance.

Single IVIG 2 g/kg/day is the current standard treatment and is recommended as class I in the AHA and JSPCCS guidelines (4, 5). During this study period, the impact of high-dose ASA on treatment has decreased (9). Recently, controversies have arisen regarding the optimal dose of ASA to be used. In 2004, the first query regarding high-dose ASA treatment was proposed. Hsieh et al., a Taiwanese group, originally performed IVIG treatment without using ASA in the acute phase, and retrospectively aggregated the antipyretic rate and the incidence of CAAs (11). Their study concluded that 94% of patients had defervescence within 3 days of IVIG treatment, and 14% had CAAs, consistent with various reports with IVIG plus ASA. In 2016, Kim et al. studied a large sample of 8,456 children from a retrospective survey of 116 hospitals in South Korea. They reported that compared with low-dose ASA (3–5 mg/kg/day), medium or higher-dose ASA (>30 mg/kg/day) for treatment of acute KD was associated with a higher risk of CAAs after adjustment for cofounders (14). In 2018, a Canadian group reported the results of a multicenter retrospective study (12). They compared the incidence of CAAs with a coronary artery Z score of ≥2.5 between high-dose aspirin (80–100 mg/kg/day) and low-dose aspirin (3–5 mg/kg/day) groups. Non-inferiority for suppression of CAAs in the low-dose group was shown. Our study in a Japanese cohort suggested non-inferiority in the ASA 30-mg Group vs. the ASA 50-mg Group. This result is consistent with a study by a Canadian group, although we used an aspirin dose difference of 20 mg/kg/day in this study.

Ogata et al. reported that 40% of KD patients had CAA with Z score ≥2.5, whereas our results indicated that 12% of patients had CAAs with Z score ≥2.5, which was much lower than the previous report. Ogata et al. collected the maximal internal diameter (mm) of the left anterior descending coronary artery and the right coronary artery within 12 weeks after onset (19). We only collected data at 1 month after onset. This may account for the low incidence of CAAs in this study.

The incidence of additional treatment in the ASA 30-mg Group was significantly higher than in the ASA 50-mg Group; the difference in incidence was 5%. Studies in Canada and South Korea have shown different incidences of 3 and 6%, respectively (12, 14). In this study, it is important to mention the high IVIG resistance rate. In our institution, non-responders were defined as those with body temperature ≥37.5°C at 48 h after initiation of primary therapy, which is the standard time point in Japan. This time point is different from that of the US, which may explain the high IVIG resistance rate. Akagi et al. and Lee et al. reported that high-dose ASA could shorten the duration of fever in their studies in a Japanese and Korean cohort, respectively (20, 21). Specifically, the duration of fever and the frequency of further treatments are not the same, but our results do not conflict with these previous reports. If the additional treatment is IVIG, the cost of medical care rises and in Japan, the facilities go into deficit. In addition, the hospitalization period is extended and the patient burden increases. Thus, 50 mg/kg/day ASA would be superior to 30 mg/kg/day ASA in terms of medical costs.

Regarding adverse events, Kuo et al. reported a decrease in hemoglobin after IVIG treatment in patients who received high-dose ASA (13). In our Japanese cohort, however, anemia did not worsen in the ASA 50-mg Group, although hemoglobin levels decreased in both groups as previously reported (22). Japanese individuals have limited hepatic metabolism of aspirin, and liver dysfunction is more frequent at the high dose of 80–100 mg/kg/day ASA. Therefore, the high dose is not approved in Japan. The incidence of liver dysfunction was similar between the two groups. There were no severe bleeding events either. In this study period, we had no patients with digestive symptoms. According to Huang et al., digestive symptoms were the most common side effects of ASA therapy (23).

There are several limitations of this study. The first limitation is selection bias in study patients. Patients who were enrolled in a clinical study (16) were excluded from the ASA 30-mg Group. The odds ratio of the ASA 30-mg Group vs. that of the ASA 50-mg Group in the incidence of CAAs was 0.769 (<1.0), whereas that in the incidence of treatment resistance was 1.379 (>1.0). Usually, the two odds ratios would be proportional. Exclusion of patients with severe disease who were enrolled in a clinical study could have influenced the primary endpoint. Second, we excluded patients who had preexisting CAAs before treatment, which was diagnosed according to the Japanese criteria (15). CAAs are underdiagnosed according to the Japanese criteria, and this study may have included patients with CAAs before treatment defined by the AHA guidelines, which diagnose CAA based on a Z score ≥2.5. Third, the aspirin dose difference between the two groups is not large. The government has approved 30–50 mg/kg/day for KD and this observational retrospective study was performed using the study design within this dose range. The 4th limitation is that this is a retrospective and single-center study.

A prospective study is currently underway in Taiwan comparing IVIG plus ASA low-dose and high-dose initial treatment (24). We are eagerly awaiting the results of that study.

In conclusion, the suppression of the development of CAAs, which is the ultimate goal of treatment for KD, might not be inferior with 30 mg/kg/day ASA compared with 50 mg/kg/day in Japanese patients. However, the administration of ASA 50 mg/kg/day could decrease the need for additional treatment without increasing adverse events.

## Data Availability Statement

All datasets generated for this study are included in the article/supplementary material.

## Ethics Statement

The studies involving human participants were reviewed and approved by Research Ethics Board, Tokyo Women's Medical University (3636-R2). Written informed consent from the participants' legal guardian/next of kin was not required to participate in this study in accordance with the national legislation and the institutional requirements.

## Author Contributions

HH and TM designed the study. HH reviewed the research and monitored ethical issues. YI analyzed data and wrote the manuscript. JT, KY, TH, and KA did critical review for the manuscript.

## Conflict of Interest

The authors declare that the research was conducted in the absence of any commercial or financial relationships that could be construed as a potential conflict of interest.
